# Intuitive Visualization and Analysis of Multi-Omics Data and Application to *Escherichia coli* Carbon Metabolism

**DOI:** 10.1371/journal.pone.0021318

**Published:** 2011-06-22

**Authors:** Brice Enjalbert, Fabien Jourdan, Jean-Charles Portais

**Affiliations:** 1 Université de Toulouse, INSA, UPS, INP, Toulouse, France; 2 INRA, UMR792 Ingénierie des Systèmes Biologiques et des Procédés, Toulouse, France; 3 CNRS, UMR5504, Toulouse, France; 4 INRA, UMR 1089 Xénobiotiques, Toulouse, France; University of Toronto, Canada

## Abstract

Combinations of ‘omics’ investigations (i.e, transcriptomic, proteomic, metabolomic and/or fluxomic) are increasingly applied to get comprehensive understanding of biological systems. Because the latter are organized as complex networks of molecular and functional interactions, the intuitive interpretation of multi-omics datasets is difficult. Here we describe a simple strategy to visualize and analyze multi-omics data. Graphical representations of complex biological networks can be generated using Cytoscape where all molecular and functional components could be explicitly represented using a set of dedicated symbols. This representation can be used i) to compile all biologically-relevant information regarding the network through web link association, and ii) to map the network components with multi-omics data. A Cytoscape plugin was developed to increase the possibilities of both multi-omic data representation and interpretation. This plugin allowed different adjustable colour scales to be applied to the various omics data and performed the automatic extraction and visualization of the most significant changes in the datasets. For illustration purpose, the approach was applied to the central carbon metabolism of *Escherichia coli*. The obtained network contained 774 components and 1232 interactions, highlighting the complexity of bacterial multi-level regulations. The structured representation of this network represents a valuable resource for systemic studies of *E. coli*, as illustrated from the application to multi-omics data. Some current issues in network representation are discussed on the basis of this work.

## Introduction

The graphical visualization and analysis of multi-omics data is a challenge in systems biology [1], [2]. The representation of true biological networks includes several layers of complexity due to the embedding of multiple biological components and processes – e.g gene expression, protein biosynthesis, regulatory processes, etc -. Given that each layer includes thousand of components even in the simplest cell, there are strong needs for visualization tools that ease the intuitive interpretation of multi-omics data. In this regard, such a tool should explicitly represent all the molecular components in the studied phenomenon (data representation), as well as all the interactions between these components (data understanding).

Numerous solutions have been recently developed for the representation of complex networks as well as the analysis of multi-omics datasets [3]. Some of the most complex tool packages (“*Cyclone*” [4], “*the Gaggle*” [5], “Prometra” [6]) succeed to present together data from multiple dimensions through the association of several software. These tools are powerful and versatile but require significant computing efforts, which can limit their use by biologists. Some commercial tools also offer a combination of data and network visualization (i.e, “Genespring”: http://www.genespring.com; “Ingenuity Pathways Analysis”: http://www.ingenuity.com/), but they are quite expensive and lack the flexibility of open-source software. Some tools have been developed to represent either metabolic or regulatory networks (e.g., *Pathway Tools* [7]), but to our knowledge, no freely-available solution has been developed to bring forward the regulatory aspect conjointly to omic-data display. Finally, the representations of biomolecular networks that are automatically generated by current software are often far from both the academic conventions and biological perception of the networks, thereby making difficult the intuitive interpretation of data.

The objective of this work was to propose a cost-less and straightforward strategy to represent both complex biomolecular networks and multi-omics data in the same graphical representation. A simple graphical formalism was designed to represent all network components (structural and functional components). These components were compiled using the open source software Cytoscape [8]. MODAM, a custom-made Cytoscape plugin, was developed to optimize the mapping of multi-omics data and their interpretation. This approach was applied to the central metabolic network of the bacterium *Escherichia coli*, as a typical example of cellular metabolism and its regulation, with hundreds of metabolic or regulatory interactions. The resulting network encompasses 774 components and 1232 interactions that are represented accordingly to biochemistry text-book drawing conventions. The mapping of multi-omics data from Ishii *et al*. [9] offered a valuable example of the approach, as discussed in the final part of this publication.

## Results

### Dedicated formalism

**Figure 1:**
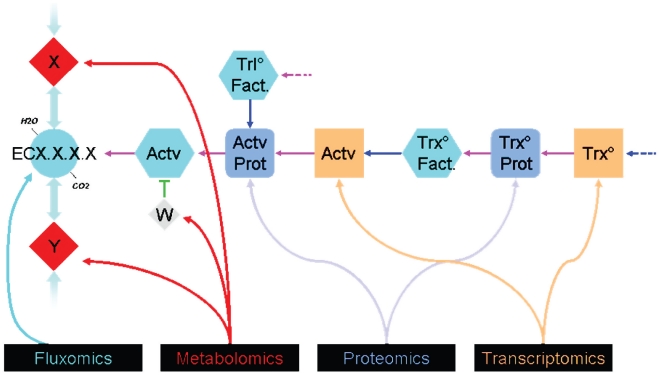
Dedicated formalism. All molecular or functional components of the metabolic and regulatory networks are explicitly represented using specific symbols. Each RNA (square) encodes a polypeptide (rounded square). Polypeptides or polypeptide complexes generate functional entities – *i.e*., enzymes or regulators – (hexagons). Enzymes catalyze reactions (circles), which allow the inter-conversion of metabolites (diamonds). A color code can be applied to each node (symbol) in the network to visualize experimental data (gene expression for the squares, protein abundance for the rounded squares, specific activity for the hexagons, metabolite concentrations for the diamonds and flux values for the circle). Interactions between the components are indicated with lines (edges). Four main kinds of interactions were considered and were represented using lines with specific colors: biochemical conversions (grey lines), transcriptional and translational regulations (blue lines), control of enzymatic activities by metabolic effectors or by phosphorylation (green lines), hierarchical relationships – i.e. RNAs to proteins, proteins to activities, activities to reactions - (pink lines). In the given example, a metabolite X is converted in Y through the reaction ECx.x.x.x. The reaction requires a molecule of H_2_O and produces a molecule of CO_2_. The metabolite W is a negative effector of this reaction. The reaction depends on the enzymatic activity “Actv” which is a property of the protein “Actv Prot”. This protein is encoded by the gene “Actv gene” whose transcription is induced by the activity “Trx° Factor”, itself resulting from the protein and gene “Trx°”. Translation of “Actv Prot” is controlled by the translation factor “Trl° Fact”.

The aim of this work was to develop a strategy for the representation of complex biomolecular networks that facilitates the intuitive interpretation of multi-omics datasets. A graphical formalism (figure 1) was introduced to represent explicitly any component of the system (RNA, proteins, activities, fluxes, and metabolites) as well as any kind of structural and regulatory interaction between two components (metabolic reactions, transcriptional and translational regulations, control of enzymes by metabolic effectors or by phosphorylation, and hierarchical relationships – genes to proteins, proteins to activities, and activities to reactions). This formalism can be applied to generate maps representing the structural and regulatory knowledge for all types of biomolecular networks. Beside its universality, the presented formalism provides a graphical representation compatible with an intuitive understanding of the network structure.

### Application to the assembly of *E. coli* central carbon metabolic network

For illustration, the graphical formalism was applied to build up a map of *Escherichia coli* central carbon metabolism and its regulations. Cellular metabolism represents a valuable example of a complex and tightly regulated biomolecular network. The central carbon metabolism is composed of a set of highly interconnected reactions that provide a variety of molecules, energy and redox power to the cell to sustain survival, growth, and adaptation. It carries some of the most basic processes of life and is subjected to intense regulation. The central carbon metabolism and its regulation have been extensively studied and a large wealth of information is available to generate a highly detailed network describing all known metabolic and regulatory interactions.

**Figure 2:**
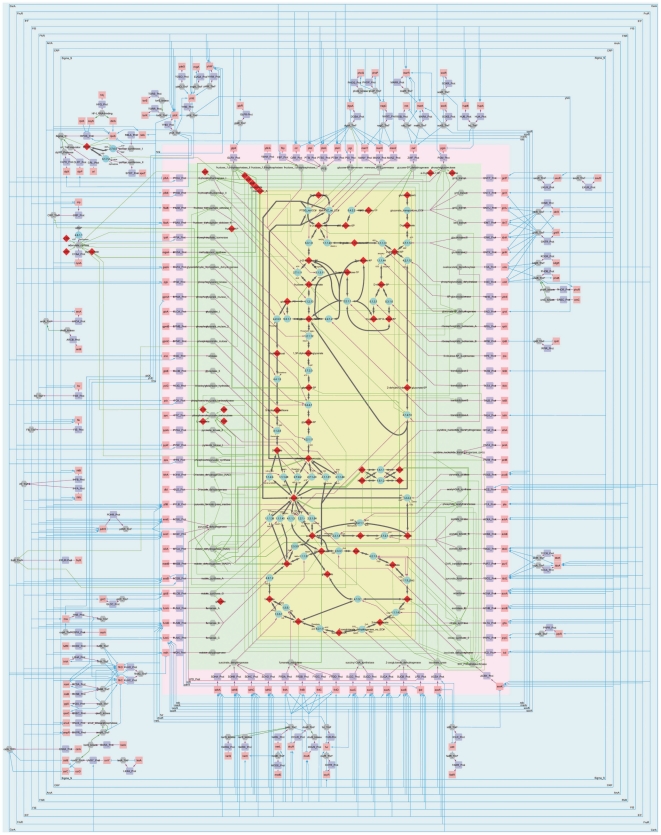
*E*. *coli* central carbon metabolism pathways and its regulations. The central part of the figure represents the central carbon metabolism (yellow background), with glucose entry (central topmost part) and gluconate entry (right topmost part), methylglyoxal pathway (leftmost central part), glycolysis/gluconeogenesis (left central part), Entner-Doudoroff pathway (rightmost central part), pentose phosphate pathway (right top part), TCA cycle with glyoxylate shunt (bottommost part), acetate metabolism (right bottom part) and transhydrogenase reactions (right central/bottom part). The first outskirt (green background) corresponds to the enzymatic activities and their metabolic controls. The second outskirt (pink background) represents the gene and protein encoding the activities. The third outskirt exhibits the direct and indirect transcriptional and traductional regulations (blue background). See the text for additional descriptions.

The first step is the network delineation, which is determined by the biologic purpose. For this work, the network has to be large enough to illustrate the strategy and consistent enough to depict *E. coli* central carbon metabolism. The network was consequently delimited to include all the central carbon metabolic pathways: glycolysis/gluconeogenesis, pentose phosphate, TCA, glyoxylate, Entner-Doudoroff, methylglyoxal and acetate (figure 2).

The second step was to collect and gather all the molecular and functional information related to this network and its regulation from relevant databases: the information was compiled for metabolic pathways (*KEGG* [10], *Ecocyc* [11]), biochemical reactions (*Brenda* [12]), and their regulations (*Ecocyc* [11], *Colibri* [13], *RegulonDB* [14]) in addition to literature data (e.g. *NCBI* [15]). Inconsistencies in the so-established network were curated using existing literature (*NCBI*) and personal expertise. The final network (figure 2 and figure S1) contained 55 metabolic reactions, 63 metabolites, and required 77 enzymatic activities generated from 93 different polypeptides and as many mRNAs. A total of 41 small molecules were identified as effectors of enzymatic activities and were responsible for 43 activations and 83 inhibitions. A total of 411 transcriptional controls were also identified. Translational controls exerted by small non-coding RNAs were also introduced where needed (*e.g.* Csr system). As most of the transcriptional factors are themselves under transcriptional control, all the indirect degrees of transcriptional regulations were included.

The third step consisted in organizing the network layout to facilitate the intuitive reading of the biological information despite the considerably high number of components. The aim is to optimize the clarity of the representation (e.g. reduction of edge lengths, etc) while respecting the academic conventions for intuitiveness. This is not feasible with the automatic tools for network representation that are currently available (figure S2). Because a metabolic-centric representation was considered, the graphical layout of *E. coli* central metabolism was designed so as to make clear the structure of the metabolic network according to usual conventions (such as a vertical glycolytic pathway from glucose at the top to a circular TCA at the bottom). Consequently, the graphical layout (figure 2) was organized as a core of metabolic processes surrounded by 3 successive layers representing respectively metabolic control (inner layer), genome expression (medium layer), and transcriptional control (outer layer, or ‘outskirt’). Some transcriptional factors (for example DgsA/Mlc, DcuR, FlhCD) were highly specific to a pathway meanwhile other factors (*e.g.* CRP, ArcA, FNR, IHF, FruR, Fis and Sigma S) were extremely pleiotropic. Likewise, ATP, ADP, AMP, phosphate and coenzyme A have numerous implications in enzymatic activity control. For these pleiotropic regulators, a “passageway” representation was introduced to avoid too many crossing lines over the central part of the graph (external blue lines, green lines surrounding the central part). The resulting interaction map provides a unique graphical interface to access the knowledge accumulated about *E. coli* central carbon metabolism and a valuable illustration of the complexity of its regulations.

It has to be noted that, using the same strategy, the same network could be represented in a different manner depending on the biological question. For illustration, it could be organised around pleiotropic transcriptional regulators if the question was mainly related to global regulation.

### Interactivity and mapping with omics data using Cytoscape

**Figure 3:**
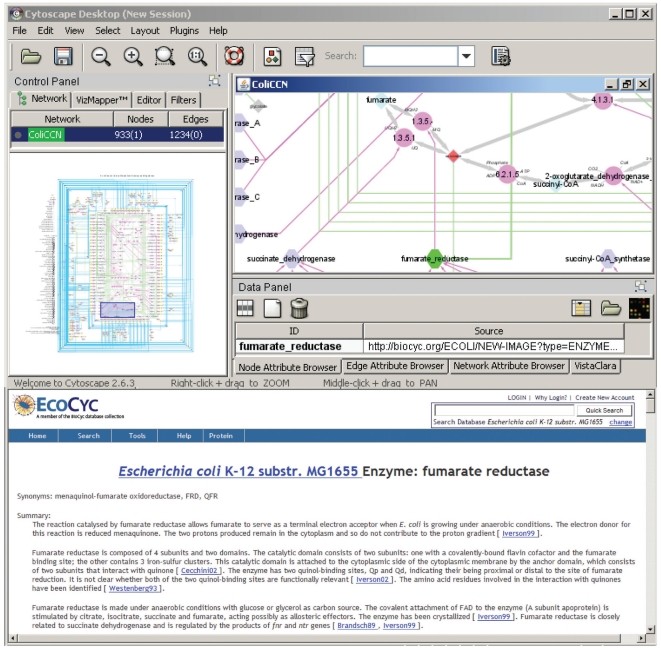
Utilization as a web-platform for biological information. Cytoscape allows linking any component of the network (node or edge) to a webpage. This functionality was used to link all the nodes and edges of the graph to relevant information in the databases from which the biological network was generated. For example, one can select the fumarate reductase activity (highlighted in green in the cytoscape main visualization window) and click on the associated link (“Source”) to access the corresponding information page in Ecocyc (bottom window).

All the relevant biological knowledge was compiled as an interactive graph object using the open source software Cytoscape [8]. Combined with the graphical formalism, this network-dedicated software was found to be a convenient and handy platform to bypass the complexity of representing multi-level regulated networks. Besides facilitating the compilation of the data in the form of a graph, it offers numerous additional benefits. A first useful functionality of Cytoscape is the possibility to link any edge or node of a graph to a specific webpage. This functionality was used to link each component of the interaction network to corresponding information web pages in relevant databases (*e.g*., *Ecocyc*, *Brenda*, *Pubmed*, etc.). In most cases, this allows getting molecule structures, reaction details, enzymatic effectors and their targets, gene and protein properties, or gene regulation networks (figure 3). Therefore, this function is extremely useful to get access to detailed information about the displayed network and its components in an interactive manner, and thereby to speed up data interpretation.

**Figure 4:**
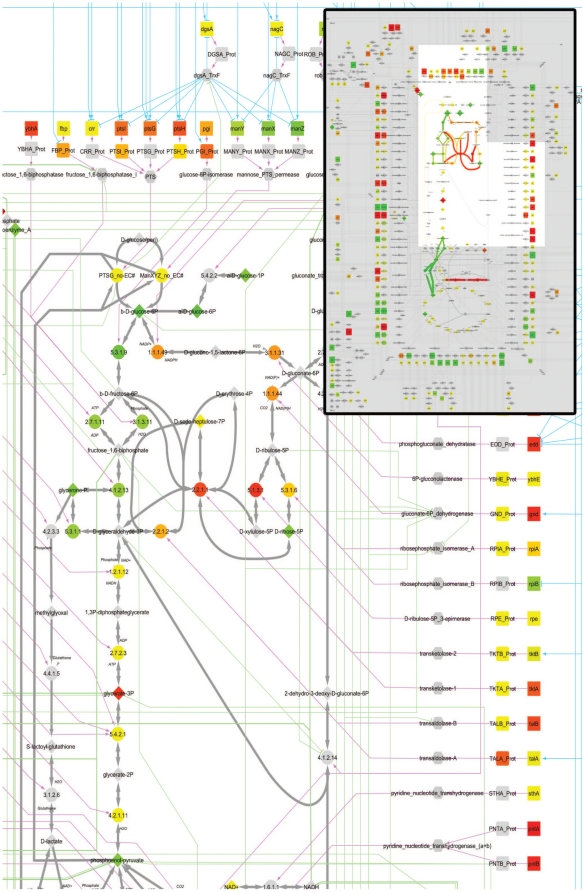
Detail of the network mapped with multi-omics data. The network was used to visualize a set of multi-omics data – including transcriptomic, proteomic, metabolomic and fluxomic data - from Ishii et al. [9]. The figure shows a detail of the whole network, which is displayed in miniature in the upper right corner with a highlight of the expanded region (using the “impact mode” while the central figure is displayed in “normal mode” ; see text for details). The displayed data correspond to the comparison of *E. coli* MG1655 K12 grown at a growth rate of 0.7 h^−1^ compared to the same strain grown at 0.2 h^−1^. The color scale tends from green for a greater value of the low growth rate to red for a greater value in the rapidly growing cells, through yellow for equivalent values. Grey shapes are nodes with no associated values. Full size of the figure in “normal mode” is available on figure S1.

In addition to graphical representation and compilation of biological information, Cytoscape offers also the possibility to map the metabolic/regulatory network with multi-omics data. Since all network components – *i.e*. RNAs, proteins, metabolites, fluxes -, are explicitly represented, the various omics data – *i.e*. transcriptomics, proteomics, metabolomics, and fluxomics – can be visualized in parallel on the same graph. A unique color scale can be applied to all nodes to plot the experimental values for all types of data. To validate this functionality, complete sets of multi-omics data extracted from the work of Ishii *et al.* [9] were plotted on the graphical display. This is illustrated in figure 4 for the comparison of two datasets corresponding to *E. coli* cells grown at µ = 0.7 h^−1^ and µ = 0.2 h^−1^, respectively. The display of the multi-omics data on the network showed the activation (in red) of the PTS gene expression at both the transcriptional and translational levels. This activation is likely to be controlled by the Mlc/dgsA transcriptional factor. Another transport system, i.e. the mannose PTS operon, is also under the control of Mlc/DgsA. However, this operon is down-regulated (green), which could be explained by the influence of the NagC transcriptional factor. In spite of the induction of the PTS transport system, the glycolytic flux seems to be stable (yellow). This apparent lack of effect is due to the fact that flux data were expressed relative to the rate of glucose uptake. Indeed, the absolute rate of glycolysis in the fast-growing cells was higher than in the slow-growing cells. In addition, the display of the relative flux data showed a significant redirection of the carbon flux towards the pentose phosphate pathway (PPP) when the growth rate is increased. The genes encoding the PPP enzymes were mostly upregulated at the transcriptional level (zwf, gnd, rpiA, rpe, tktA, talB), but not at the protein level. The correlation of transcriptomic and proteomic data showed a Pearson score of 0.04 for PPP components, compared to 0.68 for glycolysis (using the data for the four growth rates described in *Ishii et al*. [9]; data not shown). It is beyond the scope of this paper to re-interpret the authors' results but the application of the proposed formalism to this particular case does point out either inconsistencies in the data or the occurrence of an unidentified translational mechanism that controls PPP. It does however illustrate the usefulness of the introduced formalism and representation to assess the overall coherence of complex datasets and, thereby, to help formulate working hypothesis for future investigations.

### Multi-Omic Data Miner (MODAM): a Cytoscape plugin to facilitate multilayer data interpretation

Cytoscape offers the possibility to represent different entities through dedicated symbols and to apply a colour scale according to numerical values. The latter functionality is useful for the visualization of one particular type of omic data but is limited for multi-omics data representation. In particular, a graphical property (i.e. colour) can be associated to only one kind of attribute (e.g. transcriptomic values). To apply the colour scale to all omics data, all biological components must be declared with the same and unique attribute. Such generic design does not allow processing separately the different types of omics data to account for differences in data format (e.g. ratios, absolute values, etc) or differences in the amplitudes of changes between, which can be highly different from one type of data to another. The nature of changes can be different too. For instance, the flux through a reversible reaction can be orientated in the direction opposite to that of a reference condition, The reverse direction can be expressed as a negative value, and has to be explicitly visualized on the graphical representation using dedicated attributes. The application of a unique colour scale to all types of data can hamper the visibility or nature of changes associated with some types of omics data compared to other types.

**Figure 5:**
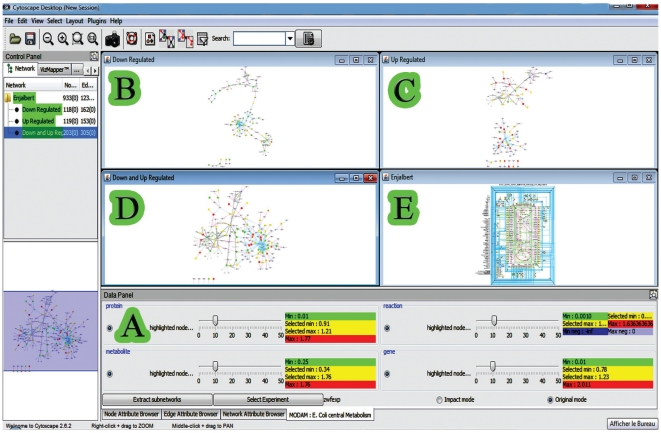
MODAM plug-in interface. The main MODAM interface is the panel below the graph representations (A). For each omics data it is possible to change the coloring threshold expressed in percentage of the significant elements. The user can also select, via the radio buttons, which omics data will be displayed and taken into account for the subnetwork extraction. Note that any modification is directly applied to the view in order to provide an interactive feedback to users. Four representations are available:,the subnetwork of down-regulated elements (B), the sub-network of up-regulated ones (C), the joint up-and down regulated elements subnetwork (D), and the global representation (E).

Here, we propose MODAM (stands for Multi-Omic Data Miner), a new plugin to overcome this problem and to extend Cytoscape functionalities toward data mining in multi-omics datasets. These functionalities are accessible through a user-friendly GUI (figure 5A). A strong benefit of MODAM is to allow multiple independent colour scales to be applied in parallel. The adjustment of the colour scale using independent cursors for each omic set is a straightforward and convenient operation. These cursors allow mapping the data according to their relative distributions and not only according to arbitrary thresholds [16]. Each individual clour scale ranges from green (ratios below one) to yellow (similar values) and red (ratios above one). Negative flux ratios were represented with a dedicated blue colour scale (see example of the PPP fluxes in figures S3 and S4) We also offer through MODAM the possibility to highlight the strongest variations by matching the node size (gene and protein expression, metabolite accumulation) or edge width (fluxes) to the fold change. This display mode (refered to as “impact mode”) is well-suited to embrace large scale network information. Both modes (“impact mode” and “normal mode”; figures S3 and S4) could be easily switched using the GUI interface of MODAM.

**Figure 6:**
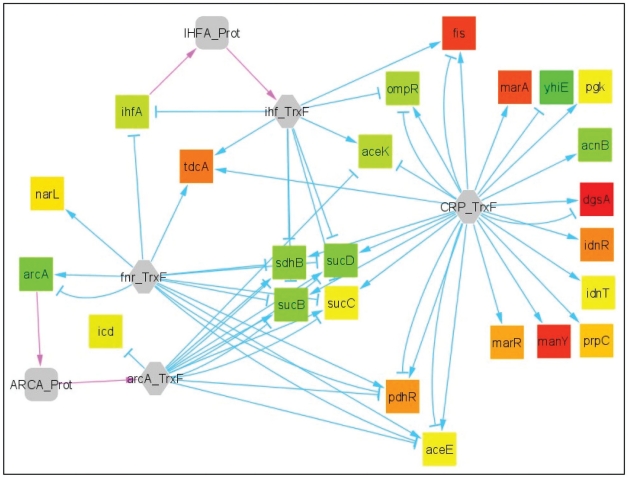
Major transcriptional factors implicated in a Δ*zwf* metabolic reshuffling using MODAM. The “select experiment” option of the MODAM interface was used to pick the Δ*zwf* data provided by Ishii *et al*. [9]. Each cursor (gene, protein, reaction and metabolite) were set on 10% of highlighted differential data. The “extract subnetworks” option was then activated to select only the differentially expressed nodes and their first neighbours (as in 5d). From the “up and down regulated” network, nodes were ranked according to their number of connections (degree). Among the nine most connected elements, four are transcription factors. The selection of these transcription factors and their neighbours was reiterated. This iterative process resulted in the presented subnetwork. The drawing was obtained using a force directed algorithm, manually adjusted to group genes that are regulated by the same sets of transcription factors (e.g. the four genes in the centre are regulated by the four transcription factors).

The complete interaction network contains hundreds of components among which many of them were not monitored by omics data or for which changes were not significant. In the biological interpretation of the results, these parts of the network can be discarded in order to focus on the core of the modifications. To do so, MODAM includes extra features like the automatic extraction of three sub-networks highlighting the most significant data. A first sub-network contains all the biological entities that are down regulated (Figure 5B). Since the components extracted by such process are not necessarily directly connected one to each other, the sub-network extraction was extended to include all their direct neighbours (*i.e.* components they are directly connected to). A second network can be obtained by extracting only up-regulated components and their neighbours (Figure 5C). Finally a third network containing both up- and down- regulated elements is proposed (Figure 5D). To illustrate, MODAM was used to extract the main changes between an *E. coli* strain (Δ*zwf*) deleted for the gene *zwf* encoding glucose-6-phosphate dehydrogenase (G6PDH) and its isogenic wild type strain [9]. The G6PDH is the first committed step of the PPP and its absence in the Δ*zwf* strain blocks the flux of carbon through the oxidative part of this pathway, resulting in significant metabolic rearrangements. The sub-network of transcriptional factors involved in the metabolic adaptation to *zwf* knock-out could be automatically extracted from the selection (Figure 5) of the most (up- and down-) regulated components. This sub-network (Figure 6) nicely highlights the role of the transcriptional factors CRP, FNR, ARCA and IHF in the resulting differential expression of central carbon metabolic genes. In particular, a pool of four less expressed genes (*sdhB*, *sucB*, *sucC*, *sucD*), all implicated in succinate metabolism, is regulated by the four transcription factors. This kind of conclusion could not be directly raised from the initial network representation since the considered components and their interactions are widely distributed over the graph.

Last but not least, the *E. coli* central carbon metabolism network presented in this work has been set up as default on the launch of MODAM, as well as the best Cytoscape parameters and visual properties to display the whole network. This will provide any user with fast access to the knowledge compiled in this work. Users can also create their own networks following the conventions given in the manual of the plugin and, this way, can apply MODAM to their own networks.

## Discussion

This work presents a simple, handy and inexpensive strategy to facilitate the visualization and analysis of complex multi-omics datasets (companion web-page at https://sites.google.com/site/modamplugin/). The central concept is to decompose the structural and functional components from gene expression to translation, from activities to fluxes. It was possible to represent the different types of interaction and therefore, to conciliate regulatory and structural networks on the same figure, and to map this network with multi-omics data. The visualization strategy could be divided in three steps: (i) network delineation and data gathering; (ii) compilation, justification and organization, using Cytoscape; (iii) data mapping and interpretation using the custom-made plugin MODAM.

The graphical representation of complex biological networks and multi-omics data is currently a challenge as many problems are known to be computationally difficult [17], [18], [19], [20]. The main difficulty lies in the design of graphical layouts that contain the complete information but facilitate data visualization and interpretation. For intuitiveness, there is likely no generic layout that can be considered since both network limits and representation depend on the biological question to be addressed. In this work, the purpose was to illustrate the potential of the visualization strategy by tackling the complexity of *E. coli* central carbon metabolism and its many regulations. Hence, metabolic processes were represented at the center of the figure and usual metabolic conventions were applied to facilitate intuitive reading. For other biological focuses (e.g. a genetic-centric purpose), different network layouts and representation conventions could be adopted for the same biomolecular network. Whatever the focus, a generic layout issue is the density of nodes and interactions to represent, which increases with the network size. The first rule is to minimize the distance between interacting nodes. However, some nodes – e.g. pleiotropic regulators in the *E. coli* network - have multiple interactions that spread over multiple sub-parts of the network. For such highly connected nodes, the ‘proximity rule’ was by-passed by creating “passageways” circling the zone of interaction. This passageway solution was very efficient to clarify the representation without loss of information or in data visualization capability. In addition to the visualization aspects, the formalism introduced here will be of interest to translate classical drawing conventions into computation. The graph structure facilitates the application of automatic mining methods (*e.g*. automatic search of highly connected genes that can correspond to important regulators) and can be exported to other bioinformatics software.

Cytoscape proved to be a highly versatile and flexible graphical platform, and met expectations as regard to the diversity of biological entities and interactions to represent. This versatility may be useful to users willing to extend the formalism introduced in this work. In terms of time, it took a couple of months for a single person to establish the *E. coli* network presented here. The extension to a different organism (the bacterium *Clostridium acetobutylicum*) required only two weeks (data not shown). For such less studied organisms, the graphical formalism could be adapted (using specific colors, size, etc) to account for uncertain information or to include data from close organisms. The proposed strategy is not limited to metabolism but can be applied to other cellular processes, e.g. signaling pathways, cellular cycle, stress responses, etc.

The main limitation was the representation and exploration of multi-omic datasets, which was overcome by developing the MODAM plugin. As shown from the examples provided in this work, MODAM greatly facilitates data mapping (intuitive graphical user interface and two display modes), allows multi-omic data representation (application of independent scales) and improves interpretation (automatic extraction of most significant information). MODAM could highlight the transcriptional factors involved in the metabolic reorganizations caused by the *zwf* deletion, which is of special interest for further comprehensive understanding of metabolic robustness. It allowed also the detection of discrepancies between transcriptomics and proteomics data in *E. coli* growing at different growth rates, rising the need for further investigations to determine whether this was due to technical issues or to the occurrence of post-transcriptional controls.

The application to the central carbon metabolism of *E. coli* proved to be a valuable illustration of the potential of the proposed visualization strategy, of its value for data assessment and mining, and for the formulation of new working hypothesis. The compilation work performed here can be freely exploited and transposed to different format/applications. In spite of the biological system complexity, the strategy does not require strong bioinformatics background and is accessible and user-friendly to users interested in omics data visualization and compilation of biological knowledge. Finally, the richness of the information displayed on a single figure as presented in this work is a first achievement and demonstrates the possibility of the approach. Taken in conjunction with current similar efforts, like the standardization of visual languages [21]; [22], this initiative can be amplified and extended from a sub-network scale to a whole-cell scale.

## Supporting Information

Figure S1 **Mapping of multi-omics data (fast growth versus slow growth) using the “normal mode”.** Plot of a full set of omics data extracted from Ishii *et al.* (2007), and corresponding to the comparison of the growth of *E. coli* at two growth rates (0.7 and 0.2 h^−1^). Data are displayed as ratios relative to the wild type. The color scale tends from green for a greater value at the low growth rate to red for a greater value in the rapid growing cells, through yellow for equivalent values. Grey shapes are nodes with no associated values.
(EPS)

Figure S2 **Automatic layout of the network using cytoscape.** Manual layout (a), cytoscape circular layout (b), hierarchical layout (c), sugiyama layout (d), spring embedded (e) and organic (f).
(EPS)

Figure S3 **Mapping of multi-omics data (** ***zwf*** **) using the “Normal mode.”** Plot of a full set of omics data extracted from Ishii et al (2007), and corresponding to the comparison of the growth of *E. coli* zwf mutant (encoding the first reaction of the pentose phosphate pathway) versus its isogenic wild type control. Data are displayed as ratios relative to the wild type. The color scale tends from green for a greater value at the low growth rate to red for a greater value in the rapid growing cells, through yellow for equivalent values. Negative fluxes are represented by a blue scale (deeper blue for stronger negative values). Grey shapes are nodes with no associated values.
(EPS)

Figure S4 **Mapping of multi-omics data (** ***zwf*** **) using the “Impact mode.”** Plot of a full set of omics data extracted from Ishii et al (2007), and corresponding to the comparison of the growth of *E. coli* zwf mutant (encoding the first reaction of the pentose phosphate pathway) versus its isogenic wild type control. Data are displayed as ratios relative to the wild type. The color scale tends from green for a greater value at the low growth rate to red for a greater value in the rapid growing cells, through yellow for equivalent values. Negative fluxes are represented by a blue scale (deeper blue for stronger negative values). Grey shapes are nodes with no associated values. Sizes of the gene, protein and metabolite nodes are adjusted depending on the fold change.
(EPS)
